# High PLA2 level is correlated with glioblastoma progression *via* regulating DNA replication

**DOI:** 10.1111/jcmm.17140

**Published:** 2022-02-15

**Authors:** Haiyun Zhang, Hanwei Zhao, Hongliang Wang, Zhongbo Yin, Kai Huang, Minhong Yu

**Affiliations:** ^1^ Department of Laboratory Medicine The Sixth People’s Hospital of Nantong Jiangsu China; ^2^ Department of Critical Care Medicine 902 Hospital of PLA Bengbu China; ^3^ Department of Orthopaedics Changshu No. 2 People's Hospital (The 5th Clinical Medical College of Yangzhou University) Changshu China; ^4^ Medical Laboratory Department, Daqing people's Hospital of Heilongjiang Province Daqing China

**Keywords:** cell cycle, DNA replication, glioblastoma, MCM2, MCM5, PLA2

## Abstract

Phospholipases A2 (PLA2) are a superfamily of enzymes, playing a critical role in the development of various human cancers. However, the mechanism of PLA2 as an oncogene in glioblastoma remains largely unknown. In this study, we explored the effects of PLA2 on glioblastoma and investigated the underlying mechanism. The results showed that PLA2 was highly expressed in glioblastoma. Patients with a high PLA2 level have low overall survival than those with low PLA2 expression. PLA2 overexpression promoted glioblastoma cell proliferation and viability and inhibited cell apoptosis by inducing cell cycle transition from G1 to S stage. Knockdown of PLA2 inhibited tumor growth in the xenograft mice model. In addition, PLA2 knockdown decreased the protein level of MCM2 and MCM5. These findings identify PLA2 as an oncogene in glioblastoma progression and provide a promising strategy to treat glioblastoma in the future.

## 1 INTRODUCTION

Glioblastoma (GBM) has been the most common and aggressive brain tumour in adults, representing a highly heterogeneous group of neoplasms that are the most challenging cancers to treat.^1^ GBM is difficult to treat due to its ability to tolerate a complex stress environment. Median overall survival of GBM patients is approximately 14 months and survive 5 survival is only 6.8%.^2^, ^3^ The current treatment options of glioblastoma are only two chemotherapies: temozolomide and bevacizumab.^4^ While the pathological diagnosis of GBM is characterized by frequent mitosis, necrosis, palisading nuclei and neovascularization, genetic studies have revealed glioblastoma to be heterogeneous and highly mutational.^5^ The features of molecular heterogeneity and mutability contribute to the poor response of glioblastoma to conventional DNA‐damaging chemotherapies. Novel therapies, drugs and drug combinations are needed to prolong GBM patient lifespan.^6^, ^7^

Phospholipase A2 (PLA2) is the name given to enzymes that catalyze the deacylation of glycerophospholipids at the sn‐2 position, producing two lipid mediators, fatty acid derivatives and lysophospholipids,^8^ and the rate‐limiting enzyme responsible for catalyzing the breakdown of phospholipids to lysophospholipids and fatty acids. PLA2 enzymes are classified into four groups based on differing molecular mechanisms, including cellular localization, substrate specificity and calcium dependence. These are as follows: secreted PLA2 (sPLA2), cytosolic PLA2 (cPLA2), calcium‐independent PLA2 (iPLA2) and platelet activating factor acetyl hydrolases. PLA2 serves as signaling molecules in certain types of malignant cancer and is associated with the initiation and progression, including prostate carcinomas, gastrointestinal and colorectal carcinomas.^9^, ^10^, ^11^, ^12^ Therefore, PLA2 has also been identified as a potential target of cancer therapy.

However, the function of PLA2 as an oncogene in GBM remains largely unknown. In this study, we mainly investigated the functions of PLA2 in GBM progression and explored the molecular mechanism. This study showed that high expression of PLA2 was correlated with low overall survival and poor prognosis. PLA2 knockdown induced the loss of MCM function by inhibiting MCM2 and MCM5 expression, leading to DNA damage, further resulting in GBM cell apoptosis increase and cell growth suppression.

## 2 MATERIALS AND METHODS

### 2.1 Tissue samples from patients with GBM

PLA2 expression between tumour and normal tissues of GBM was analyzed from the Sixth People's Hospital of Nantong. A total number of 11 GBM tissues and adjacent normal tissues were obtained from the Sixth People's Hospital of Nantong. Informed consent was signed by all the patients. Tissues were frozen in liquid nitrogen and stored at −80°C before experiments. All samples were collected in accordance with ethical guidelines, and written informed consent was received. All patients were approached based on the approved ethical guidelines, and patients who agreed to participate in this study were required to sign consent forms before being included in the study. All experimental protocols and methods were approved by the Medical Ethical committee of the Sixth People's Hospital of Nantong No 20210622.

### 2.2 Cell lines and culture

Glioblastoma cell lines (SHG‐44, U251 and SNB‐19) and one normal glial cell (HEB) were purchased from ATCC (American Type Culture Collection) and cultured in RPMI1640 (Gibco; Invitrogen) supplemented with 10% FBS (fetal bovine serum; GIBCO, USA). All these were maintained in an atmosphere incubator at 37°C and 5% CO_2_.

### 2.3 Cell transfection

To overexpress PLA2, glioblastoma cell lines were transfected using the pCDNA3.1(+) vector. By using small interfering RNAs (siRNAs) and short hairpin RNAs (shRNAs), RNAi‐mediated ablation of endogenous PLA2 was induced. SiPLA2, shPLA2, pcDNA3.1‐PLA2 and pcDNA3.1‐vector were transfected into SNB‐19 or SHG44 cells by Lipofectamine 2000 (Thermo Fisher Scientific).

### 2.4 Western blotting assay

Total protein was extracted from glioblastoma cells and tissues using lysis RIPA buffer containing phosphatase and protease inhibitors. After that, 10% sodium dodecyl sulfate (SDS)‐polyacrylamide gels were used to separate the protein. Then, these proteins were transferred to PVDF membranes at 250 mA for 2 h. The membranes were blocked with 5% skim milk at room temperature for 1 h. Subsequently, these membranes were incubated at 4°C for 16 h with antibodies targeted against PLA2, GAPDH, MCM2, MCM5 and γ‐H2AX. Then, they were washed with TBST and incubated with secondary antibodies. Protein signals were visualized using an ECL kit (Millipore).

### 2.5 Colony formation assay

Cells were seeded in 6‐well culture plates for 2 weeks. Individual colonies were fixed and stained with a solution containing 0.2% crystal violet in 10% ethanol for 30 min. Colony images were taken after washing and air‐drying.

### 2.6 MTT assay

Cells were seeded in 6‐well plates and incubated overnight. Then, cell viability was analyzed by the MTT assay according to the manufacturer's instructions. The absorbance at a wavelength of 450 nm was measured for the supernatant of each well using the plate reader Multiskan EX (Thermo Fisher Scientific Inc.,).

### 2.7 Cell cycle analysis

Cells were fixed with 70% ethanol overnight at 4°C. After being washed with phosphate‐buffered saline, the cells were stained with RNase A/PI for 30 min at 37°C. After that, the cell cycle phase was detected by using a flow cytometer.

### 2.8 Caspase 3/7 activity analysis

After transfection, the cells were seeded in 96‐well plates and then collected and lysed by lysis buffer. The supernatant fluid was obtained after centrifugation. Reaction buffer and caspase 3/7 substrate was mixed with the supernatant fluid. After that, the 405 nm absorbance value was measured by the SpectraMax M3 microplate reader.

### 2.9 Animal experiments

Ten female Balb/c nude mice were obtained. All animal experiments followed the guidelines of animal research. StableSHG‐44 cells with PLA2 knockdown were injected into the right oxters of five mice individually. The remaining were injected with SHG‐44cells as the control group. Tumour volume was detected every 3 days by using a digital caliper. After 24 days, the tumour weight was measured after mice were sacrificed.

### 2.10 Statistical analysis

Data are presented as mean ± standard error of the mean (SEM). Statistical analysis was performed with SPSS software. Data analysis was performed by using two‐tailed Student's‐tests. *p* < 0.05 was regarded statistically significant.

## 3 RESULTS

### 3.1 PLA2 was highly expressed in GBM

**FIGURE 1:**
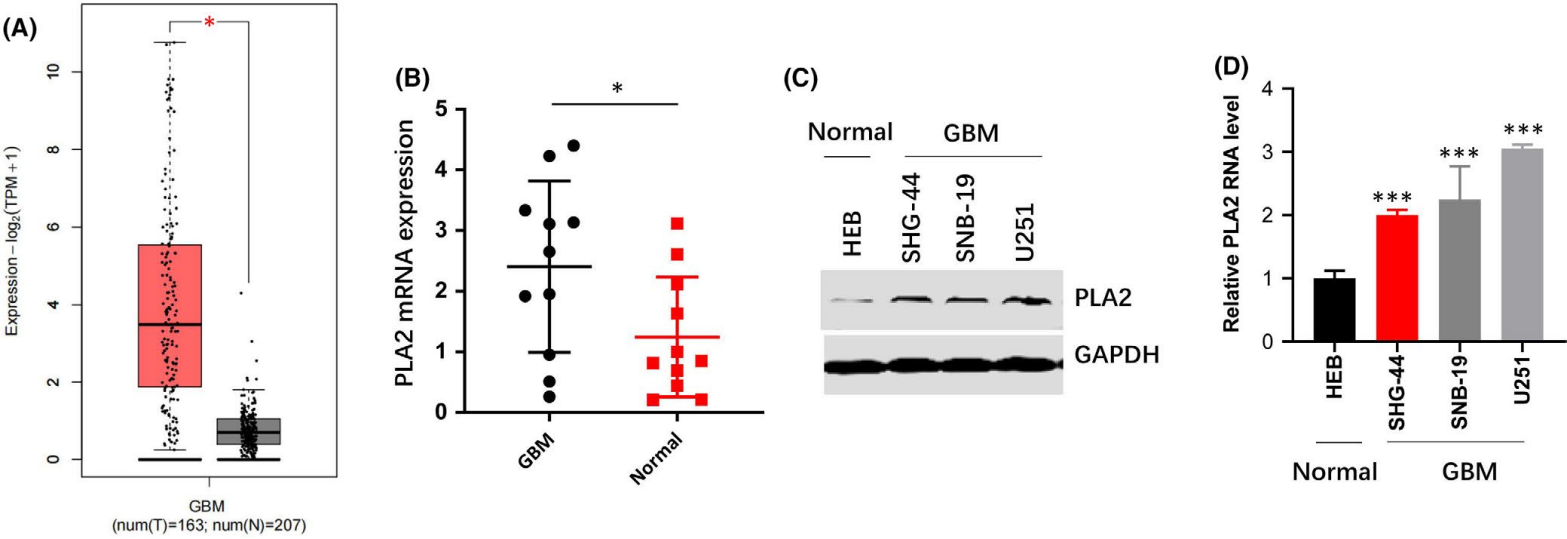
PLA2 was highly expressed in GBM. A. Multiple Oncomine analyses were performed using published datasets to examine PLA2 levels in human GBM. B. PLA2 was highly expressed in tumour tissues than with normal tissues. C. Western blotting and q‐PCR assay showed that PLA2 levels in normal cell lines (HEB) were lower than those in glioblastoma cell lines (SHG‐44, SNB‐19, and U251). Data represent mean ± S.E.M. *, *p* < 0.05

To explore the functions of PLA2 in glioblastoma, we used multiple Oncomine analyses to detect the expression of PLA2 based on published datasets. Interestingly, an increased expression of PLA2 was defined in glioblastoma tissue relative to normal tissue (Figure 1A). In accordance with this bioinformatics result, PLA2 was highly expressed in tumour tissues compared with adjacent normal samples (Figure 1B). Next, three glioblastoma cell lines, namely, SHG‐44, U251 and SNB‐19 and one human normal glial cell line, HEB, were used. Increased PLA2 productions were observed in glioblastoma cells compared with normal cells (Figure 1C,1D), suggesting that PLA2 expression might play an oncogenic role in glioblastoma development.

### 3.2 PLA2 promoted cell proliferation and viability

**FIGURE 2:**
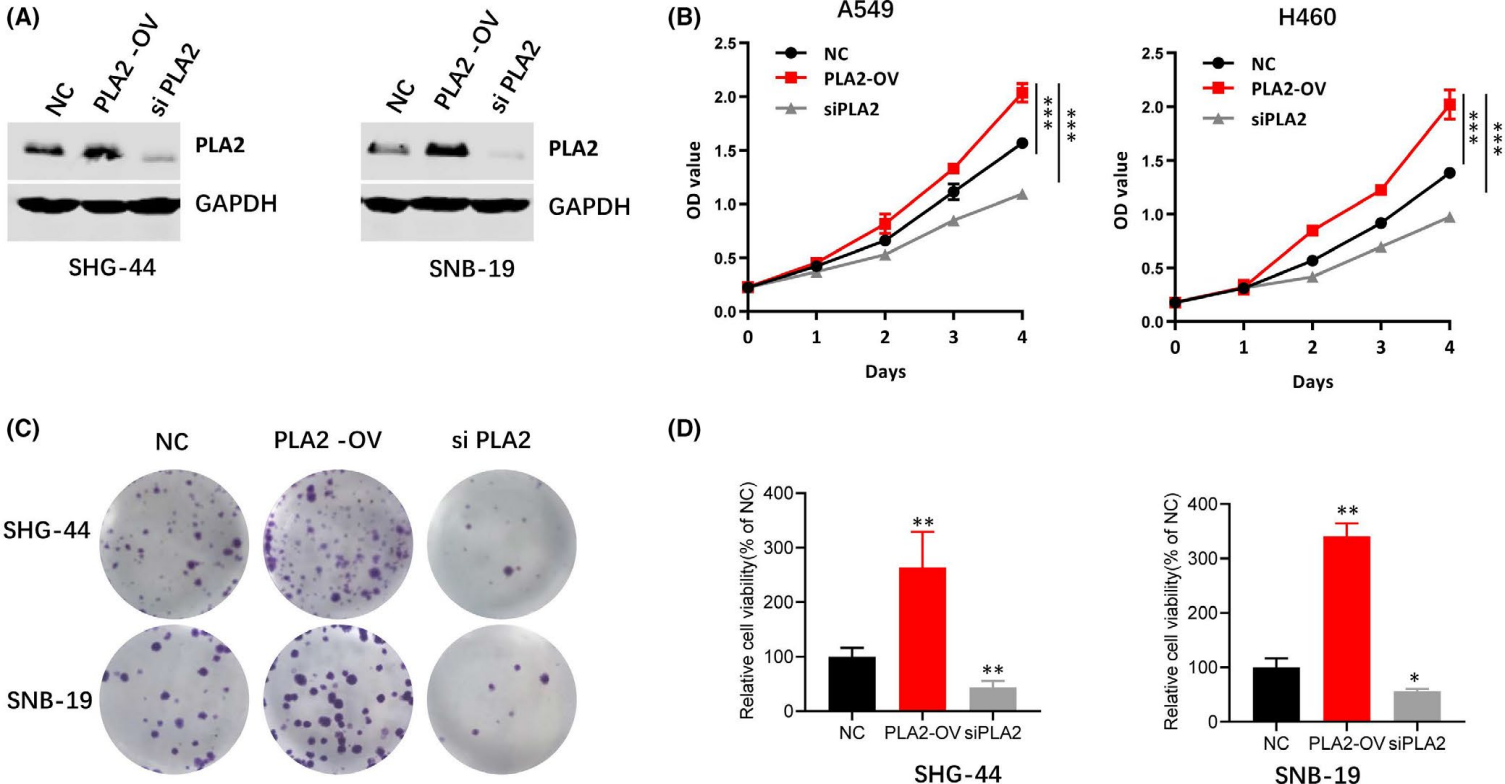
PLA2 promoted cell proliferation and viability. A. Stable cell lines with PLA2 overexpression (PLA2‐OV) or knockdown (siPLA2) were constructed, and the protein levels of PLA2 were validated by Western blotting assay. B. Overexpression of PLA2 significantly promoted cell growth in SHG‐44 (A) or SNB‐19 (B) cell lines, and PLA2 silencing inhibited cell growth. C–D. Overexpression of PLA2 significantly promoted cell viability. On the contrary, PLA2 blocking significantly inhibited cell viability. NC: normal control; PLA2‐OV: cells with PLA2 overexpression; siPLA2: cells with PLA2 knockdown. Data represent mean ± S.E.M. *, *p* < 0.05. **, *p* < 0.01. ***, *p* < 0.001

To explore the effect of PLA2 on the proliferation of glioblastoma cells, SHG‐44 and SNB‐19 cells were transfected with si PLA2 or pcDNA3.1‐PLA2 to establish PLA2 knockdown or overexpression cells. The protein levels of PLA2 in these established cells were verified using Western blotting assay (Figure 2A). MTT assay and colony formation assay were further used to detect cell proliferation and viability. The OD value of cells with PLA2 overexpression was significantly higher than that of control cells, whereas the value was decreased in cells with PLA2 knockdown compared with that of control cells (Figure 2B). In addition, PLA2 overexpression caused the increase of glioblastoma cell viability, and PLA2 silencing suppressed this process (Figure 2C and 2D). Therefore, these findings indicate that PLA2 promoted cell proliferation and viability.

### 3.3 PLA2 promoted cell cycle transition from G1 to S stage and inhibited cell apoptosis

**FIGURE 3:**
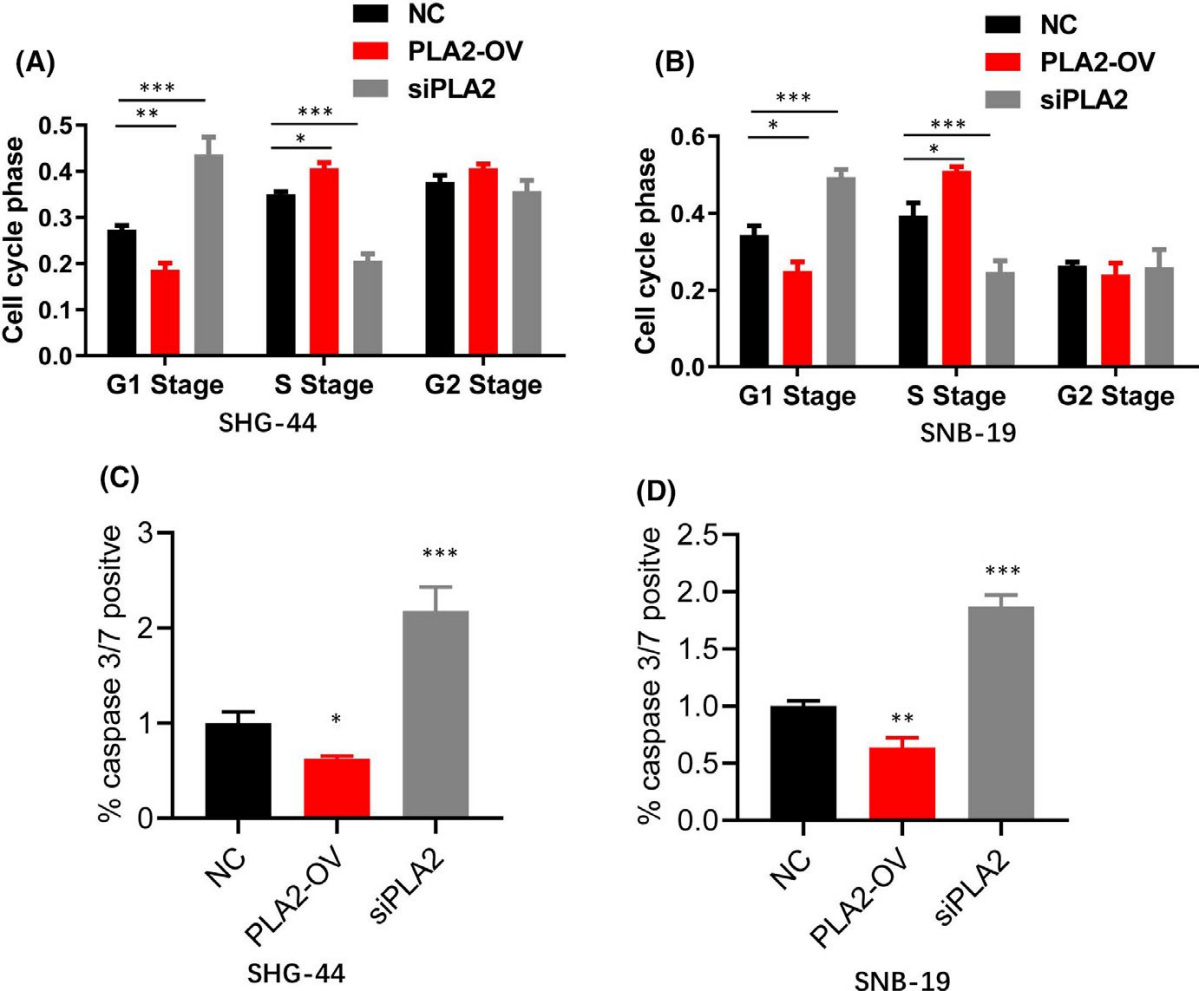
PLA2 induced cell cycle transition from G1 to S stage and inhibited cell apoptosis. A–B. PLA2 overexpression promoted G1–S transition in both SHG‐44 and SNB‐19 cell lines, while knockdown of PLA2 caused cell cycle arrest at the G1 stage. C–D. PLA2 overexpression significantly decreased caspase 3/7 activity, while PLA2 knockdown increased its activity both in SHG‐44 and SNB‐19 cell lines. NC: normal control; PLA2‐OV: cells with PLA2 overexpression; siPLA2: cells with PLA2 knockdown. Data represent mean ± S.E.M. *, *p* < 0.05. **, *p* < 0.01, ***, *p* < 0.001

By using FCM, cell cycle analysis was performed. Overexpression of PLA2 induced cell cycle transition from G1 to S stage, and PLA2 knockdown caused cell cycle arrest at the G1 stage. However, there was no significant difference for the G2 stage in SHG‐44 and SNB‐19 cells (Figure 3A–3B).

Caspase 3/7 is always used as a valuable biomarker for cell apoptosis. Therefore, we can evaluate the cell apoptosis level by testing positivity of caspase 3/7. The results showed that PLA2 knockdown significantly increased caspase 3/7 activity both in SHG‐44 and SNB‐19 cells, while PLA2 overexpression inhibited its activity (Figure 3C–3D). Therefore, these findings suggest that PLA2 promoted cell proliferation and inhibited cell apoptosis by facilitating G1/S cell cycle transition.

### 3.4 PLA2 increased MCM2 and MCM5 expression, and decreased γ‐H2AX protein level

**FIGURE 4:**
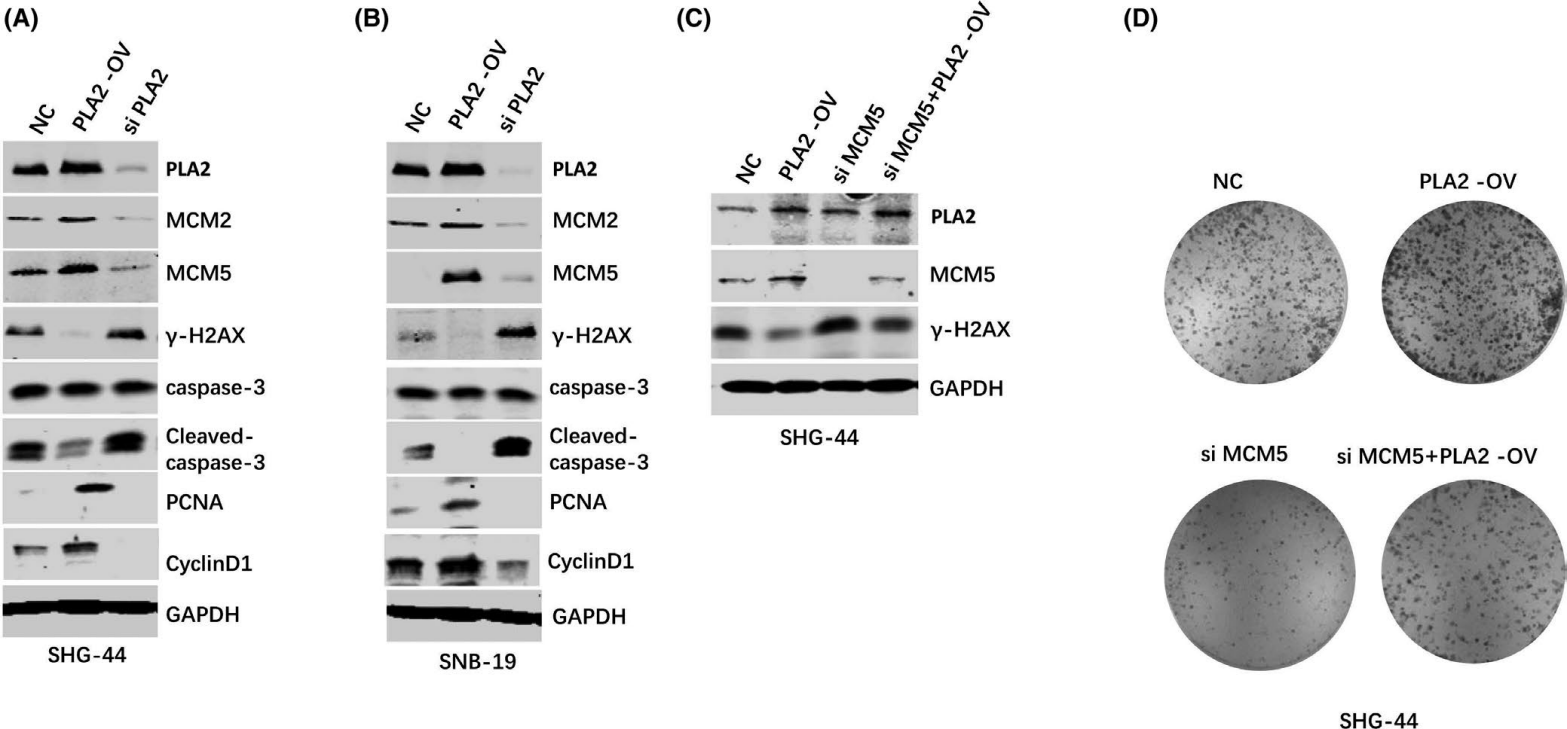
PLA2 increased MCM2/MCM5 protein levels and decreased the γ‐H2AX protein level. A–B. Western blotting assay showed the protein levels of MCM2, MCM5, caspase‐3, PCNA, CyclinD1 and γ‐H2AX in SHG‐4 SHG‐44 (A) or SNB‐19 (B) cell lines with PLA2 overexpression or PLA2 silencing. C. Expressions of MCM5 and γ‐H2AX were examined by Western blotting before and after MCM5 knockdown in PLA2 overexpressed SHG‐44 cells. D. Colony formation assay showed a rescue phenomenon after MCM5 knockdown. NC: normal control; PLA2‐OV: cells with PLA2 overexpression; siPLA2: cells with PLA2 knockdown

This study found that PLA2 induced G1/S cell cycle transition. It is well‐known that G1 and S stage are involved in DNA replication. Recent evidence supports PLA2 participation in the cell replication machinery by associations at DNA replication origins.^13^, ^14^ Thus, this study further investigated the role of PLA2 in the regulation of MCM2, MCM5 and γ‐H2AX expressions. MCM2 and MCM5 are reported to be the essential factors to regulate the initiation of DNA replication. γ‐H2AX is the biomarker for DNA damage.^15^, ^16^ The results showed that both in SHG‐44 and SNB‐19 cell lines, overexpression of PLA2 increased the protein levels of MCM2 and MCM5. Meanwhile, silencing PLA2 caused the reduction of MCM2 and MCM5 compared with normal group. For γ‐H2AX, the results were reverse. The γ‐H2AX protein level was lower in cells with PLA2 knockdown and higher in cells with PLA2 overexpression than with the normal group (Figure 4A–4B). To determine whether PLA2‐γ‐H2AX correlation was MCM5‐dependent, we depleted MCM5 in PLA2 overexpressed SHG‐44 cells using lentivirus stably expressing MCM5‐siRNA. Silencing MCM5 significantly increased expression of γ‐H2AX in PLA2 overexpressed cells (Figure 4C). Furthermore, the colony formation assay also showed a rescue phenomenon after knockdown of MCM5 (Figure 4D). These findings suggest that blocking PLA2 inhibits DNA replication initiation‐related genes, MCM2 and MCM5, thus, inducing DNA damage.

### 3.5 PLA2 knockdown decreased glioblastoma growth in the xenograft mouse model

**FIGURE 5:**
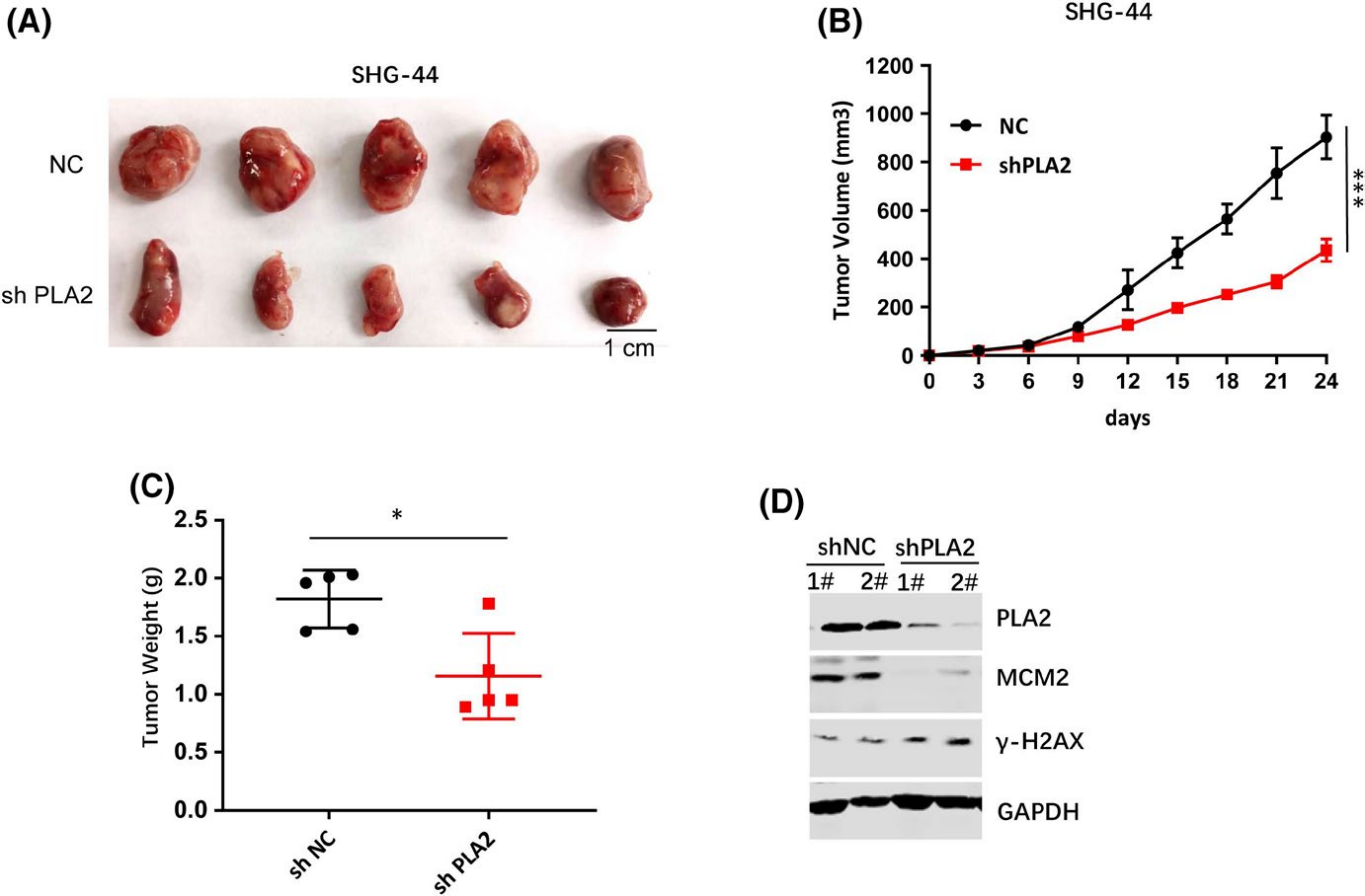
PLA2 silencing inhibited tumour growth in vivo. A. A stable PLA2‐knockdown (shPLA2) SHG‐44 cell line was subcutaneously injected into 6‐week‐old immunocompromised mice. At the end of the assay, tumours were removed and photographed. B. Tumour volume was measured every three days. PLA2 knockdown inhibited tumour volume. C. Knockdown of PLA2 inhibited tumour weight. D. PLA2 silencing caused the decrease of the MCM2 protein level and the increase of γ‐H2AX in tumor tissues. Data represent mean ± S.E.M. *, *p* < 0.05. ***, *p* < 0.001

To further investigate the effect of PLA2 on glioblastoma growth in vivo, SHG‐44cells transfected with sh PLA2 were injected into the flank region of nude mice subcutaneously. Tumour volume was measured every three days. After 24 days, mice were sacrificed, and tumour tissues were obtained. The result in vivo was consistent with that in vitro. PLA2 knockdown significantly inhibited tumour volume and weight compared the control group (Figure 5A–5C). In addition, PLA2 knockdown suppressed MCM2 expression and increased the γ‐H2AX protein level in tumour tissues (Figure 5D).

## 4 DISCUSSION

The phospholipase A_2_ (PLA_2_) superfamily contains more than 50 enzymes in mammals that are subdivided into several distinct families on a structural and biochemical basis. It plays an important role in cancer cell proliferation, invasion and metastasis, and also is used as a biomarker for cancer diagnosis or progression evaluation. An increasing body of evidence supports the excessive activation of PLA2 in many cancers, such as lung cancer, hepatocellular carcinoma, osteosarcoma and glioma as well.^17^ However, the function of PLA2 as an oncogene in GBM remains largely unknown. Thus, this study explored the effects of PLA2 on glioblastoma development and the underlying mechanism. The results proved that PLA2 played an oncogenic role in glioblastoma.

In detail, this study found that PLA2 was highly expressed in GBM tissues and cell lines. The high expression level of PLA2 was correlated with poor prognosis in patients with GBM. PLA2 proves effective for the diagnosis and pathological classification of lung cancer. PLA2 triggers cell cycle arrest in cancer cells, and apoptosis is the predominant cell death mode in PLA2‐induced cytotoxicity. Subsequently, knockdown of PLA2 suppressed cell proliferation in vitro and tumour growth in vivo. Moreover, PLA2 silencing induced G1/S cell cycle arrest and cell apoptosis in SHG‐44 and SNB‐19 cells, whereas, PLA2 overexpression leads to opposite results.

It is well‐known that G1 and S stages are linked to DNA replication. Recent evidence supports HOX proteins participation in the cell replication machinery by associations at DNA replication origins.^18^, ^19^, ^20^ PLA2 was reported to be implicated in DNA replication.^13^, ^14^, ^21^ We hypothesize that PLA2 may be involved in glioblastoma progression by regulating replicative complex assembly of DNA replication origins. Thus, this study evaluated the protein levels of MCM2 and MCM5. The mini‐chromosome maintenance protein (MCM) family members are highly conserved replication initiation factors, playing an important role in DNA replication by cyclical DNA‐unwinding. MCM2‐7, a conserved set of six related proteins, is a target of S‐phase checkpoints, and their function is required for processive DNA replication throughout S phase.^22^ Loss of MCM function during S phase generates chromosome instability and DNA damage. MCM2 and MCM5 were integrated into pre‐replicative complexes formed during the G1 stage of the cell cycle.^23^, ^24^, ^25^ In this study, PLA2 overexpression increased MCM2 and MCM5 protein levels, whereas PLA2 knockdown inhibited their expression in glioblastoma. MCM2 and MCM5 are the key components of the MCM complex. Low expression of MCM2 and MCM5 caused by PLA2 silencing leads to the loss of MCM function, resulting in DNA damage. Thus, we further investigate the protein level of γ‐H2AX, a marker of DNA damage. PLA2 knockdown decreased the γ‐H2A protein level in glioblastoma, suggesting that PLA2 blocking caused DNA damage. It was found that high levels of PLA2 repaired DNA damage more efficiently and resumed transcription and growth in breast cancer; while their low‐PLA2 expressing counterparts eventually committed to apoptosis.^21^ PLA2 is reported to be involved in driving checkpoint recovery after DNA damage, a key pathway that allows cancer cells to overcome damage response arrest.^14^ These reports indirectly confirmed our point that PLA2 knockdown induced the loss of MCM function by inhibiting MCM2 and MCM5 expression, leading to DNA damage, further resulting in glioblastoma cell apoptosis increase and cell growth suppression.

## 5 CONCLUSION

In this study, elevated expression of PLA2 is correlated with low overall survival and poor prognosis. PLA2 knockdown inhibits glioblastoma cell proliferation in vitro and tumour growth in vivo, and accelerates cell prognosis by inducing G1/S cell cycle arrest. In addition, PLA2 knockdown induces the loss of MCM function by inhibiting MCM2 and MCM5 expression, leading to DNA damage. These findings identify PLA2 as an oncogene in glioblastoma progression and provide a promising strategy to treat glioblastoma in the future.

## CONFLICTS OF INTEREST

The authors declare that they have no potential competing interests.

## AUTHOR CONTRIBUTION

**haiyun zhang:** Investigation (equal); Resources (equal); Writing – review & editing (equal). **Hanwei Zhao:** Conceptualization (equal); Investigation (equal); Resources (equal). **Hongliang Wang:** Conceptualization (equal); Data curation (equal). **Zhongbo Yin:** Resources (equal); Writing – original draft (equal). **Kai Huang:** Investigation (equal); Writing – original draft (equal); Writing – review & editing (equal). **Minhong Yu:** Funding acquisition (equal); Supervision (equal); Writing – review & editing (equal).

## Data Availability

The data used to support the findings of this study are included within the article.
